# Take Life Easy

**Published:** 1859-11

**Authors:** 


### TAKE LIFE EASY.

"*How old they look!*" said the celebrated Indian chief, *Kahgegaboo*, on his return to New York, after a comparatively short absence. Young men, whom he had been accustomed to meet daily, seemed to have grown old in four or five years. Faces which formerly appeared never to have known what anxiety was, had, in so short a time, grown care-worn, and an expression of mingled sadness and solicitude pervaded almost every countenance.

The observation was founded in fact. Our young men do grow old before their time, in their torturing haste to obtain a millionarity. Not one in a multitude has the stern courage to adopt practically the sage motto, *festina lente.* Few seem to appreciate the wisdom of making haste slowly. Few indeed have that cheerful satisfaction in moderate daily gains which so much contributes to the every day enjoyment of life. Yet if we take the pains to look over the past, we shall scarcely fail to find that men of moderate ambition, satisfied with a slow and steady increase to their fortunes, who set their face, as a flint, against every enterprise promising splended dividends, have come to a quiet old age, having enjoyed their affluence for a young life time. On the other hand, of those who boldly embarked in the "*splendid speculation*," risking all on the hazard of a die—how have multitudes of them gone out in the night of a drunkard's grave, or having made a wreck of position and character, have ended life early, in dishonorable obscurity, or in pinching penuary!

And of the young who joined in the race of ambition, who dashed madly after professional distinction, mounting rapidly to the skies with a meteor-like effulgence—how many of them too have fallen suddenly from their high estate, and left a worse than midnight gloom hanging around their memories!

In view of the whole subject, we cannot do better than close the article, as we began it, with the words of another celebrity, *Professor Silliman:*

"YOUNG MEN! *do not use up all your capital in the beginning of life.*"

